# Supplementary material for: Kaempferol and Biomodified Kaempferol from Sophora japonica Extract as Potential Sources of Anti-Cancer Polyphenolics against High Grade Glioma Cell Lines
Source: Int J Mol Sci. 2023 Jun 27;24(13):10716. doi: 10.3390/ijms241310716 (PMC10341967; doi:10.3390/ijms241310716)
Supplement: Supplementary file 1 [file ijms-24-10716-s001.zip › ijms-2142753-supplementary.pdf]

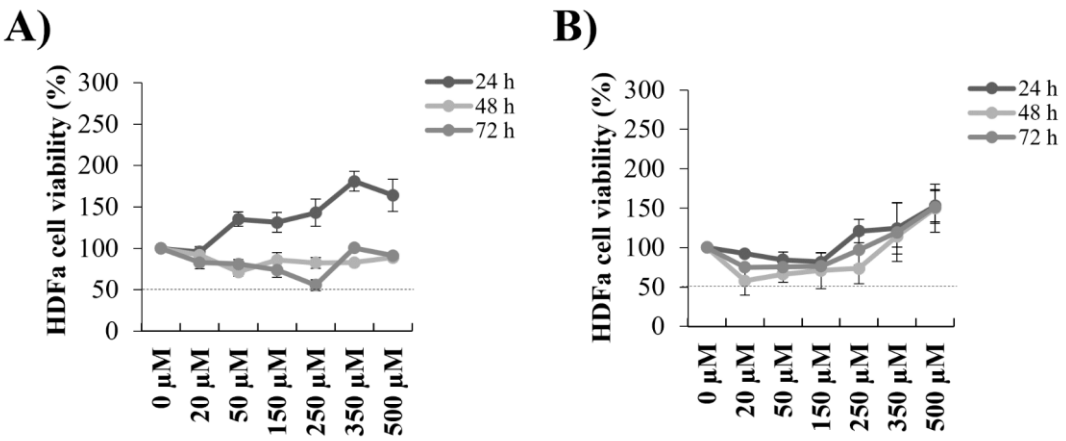

Suplemmentary Figure S1. Kaempferol (KPF-BBR) and Biomodified Kaempferol (KPF-ABR) reduces cell viability on non-tumoral human dermal fibroblast cell line (HDFa) cell line. Normal cells were treated with different concentrations of (A) KPF-BBR (left side) or (B) KPF-BBR (right side) for 24, 48 and 72 hours and further were evaluated by MTT assay. It was observed that cell viability, regardless time and concentrations, remained above 50%.

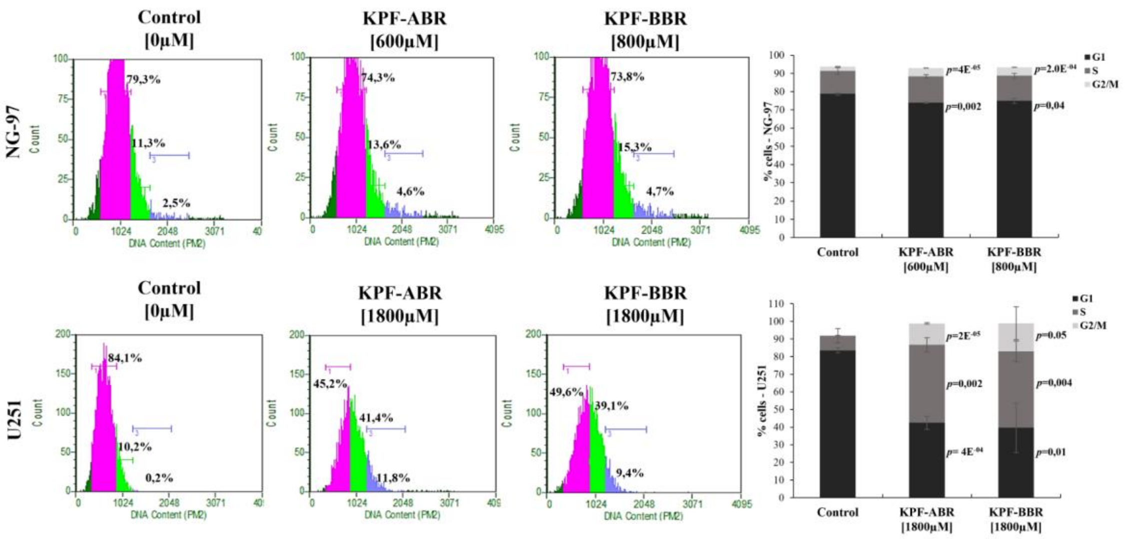

Supplementary Figure S2. Kaempferol (KPF-BBR) and Biomodified Kaempferol (KPF-ABR) induce cell cycle arrest in gliomas cells. Representative results showing a significant percentage of cells in G1 and G2/M phases in NG-97 and G1, S, and G2/M arrest in U251 cell line by flow cytometry. The top Histogram shows the mean percentage of G1 (78.80%, 74.00%, and 74.93%), S (12.5%, 14.37%, and 13.83%), and G2/M (2.45%, 4.53%, and 4.57%) in control NG-97 cells compared to KPF-ABR and KPF-BBR-treated cells, respectively. In addition, bottom histogram shows the average

percentage of G1 (83.50%, 42.55%, and 39.54%), S (8,30%, 44.20%, and 43.30%), and G2/M (0.17%, 12.10%, and 16.00%) in control U251 cells compared to KPF-ABR (0% vs. 42.55%) and KPF-BBR-treated cells, respectively. The data presented are the mean ± standard deviation of the experiments performed in triplicate.
